# Proteome dynamics of cold-acclimating *Rhododendron* species contrasting in their freezing tolerance and thermonasty behavior

**DOI:** 10.1371/journal.pone.0177389

**Published:** 2017-05-23

**Authors:** Jose V. Die, Rajeev Arora, Lisa J. Rowland

**Affiliations:** 1 Agricultural Research Service, U.S. Department of Agriculture, Beltsville, Maryland, United States of America; 2 Department of Horticulture, Iowa State University, Ames, Iowa, United States of America; Iwate Daigaku, JAPAN

## Abstract

To gain a better understanding of cold acclimation in rhododendron and in woody perennials in general, we used the 2D-DIGE technique to analyze the rhododendron proteome during the seasonal development of freezing tolerance. We selected two species varying in their cold acclimation ability as well as their thermonasty response (folding of leaves in response to low temperature). Proteins were extracted from leaves of non-acclimated (NA) and cold acclimated (CA) plants of the hardier thermonastic species, *R*. *catawbiense* (Cata.), and from leaves of cold acclimated plants of the less hardy, non-thermonastic *R*. *ponticum* (Pont.). All three protein samples (Cata.NA, Cata.CA, and Pont.CA) were labeled with different CyDyes and separated together on a single gel. Triplicate gels were run and protein profiles were compared resulting in the identification of 72 protein spots that consistently had different abundances in at least one pair-wise comparison. From the 72 differential spots, we chose 56 spots to excise and characterize further by mass spectrometry (MS). Changes in the proteome associated with the seasonal development of cold acclimation were identified from the Cata.CA—Cata.NA comparisons. Differentially abundant proteins associated with the acquisition of superior freezing tolerance and with the thermonastic response were identified from the Cata.CA—Pont.CA comparisons. Our results indicate that cold acclimation in rhododendron involves increases in abundance of several proteins related to stress (freezing/desiccation tolerance), energy and carbohydrate metabolism, regulation/signaling, secondary metabolism (possibly involving cell wall remodeling), and permeability of the cell membrane. Cold acclimation also involves decreases in abundance of several proteins involved in photosynthesis. Differences in freezing tolerance between genotypes can probably be attributed to observed differences in levels of proteins involved in these functions. Also differences in freezing tolerance may be attributed to higher levels of some constitutive protective proteins in Cata. than in Pont. that may be required to overcome freeze damage, such as glutathione peroxidase, glutamine synthetase, and a plastid-lipid-associated protein.

## Introduction

Cold stress is a major environmental factor that limits plants’ survival and geographical distribution [1]. Plants from temperate regions are chilling tolerant (0 - ~15°C) and, while most have some ‘constitutive’ freezing tolerance (FT; <0°C), they typically have a genetic ability to increase their FT when exposed to inductive environmental conditions via a process called cold acclimation [2]. Numerous studies investigating the cellular/molecular mechanisms of cold acclimation point to a multi-genic nature of this phenomenon marked by: membrane modifications, accumulation of specific proteins, lipids, carbohydrates, compatible solutes, hormones, and/or secondary metabolites, changes in cell/tissue-hydration status and cell wall structure, and cytosolic calcium fluxes [3].

A large majority of cold acclimation studies has focused on herbaceous species such as cereal grasses, potato, alfalfa, and, more recently and extensively, *Arabidopsis*. Woody perennials, on the other hand, have received far less attention in this context. In contrast to herbaceous species with moderate ability to cold acclimate (acclimated FT reaching up to ~ -20 - -30°C), many woody species can survive some of the lowest temperatures on Earth for extended periods and can acclimate down to colder than -50°C [4–5]. Conceivably, molecular mechanisms underlying the acquisition of such extreme FT may be qualitatively different from those employed by the hardy herbaceous species [6].

Woody perennials have several physiological traits that confound FT or cold acclimation research. First, tissues within an overwintering plant can exhibit different FT mechanisms, i.e. deep supercooling (avoidance of freeze-desiccation) in xylem parenchyma and bud meristems *versus* equilibrium freezing (tolerance of freeze-desiccation) in leaf and bark tissues [7]. Second, some woody plant tissues (e.g., buds) during the annual growth cycle undergo endodormancy and cold acclimation simultaneously since both are regulated by low, non-freezing temperatures and/or short days [8–10]. This superimposition renders it difficult to specifically discern the molecular/metabolic changes associated with the regulation of endodormancy *versus* cold acclimation. A few studies focused on identifying cold acclimation-specific proteins have addressed this dilemma by using strategies to physiologically de-link the changes in FT from those in endodormancy [10–12].

Aside from the commercial value as landscape plantings, *Rhododendron* genus has several attributes making it an exemplar to gain insight into the biology of cold acclimation in a woody perennial. Over 800 species of *Rhododendron* are distributed throughout the Northern Hemisphere including those in section *Ponticum* that are broadleaf evergreens with leaves withstanding freezing temperatures as low as -40° to -60°C [13]. Use of overwintering leaves in cold acclimation studies allows circumventing the problem of overlapping endodormancy as is encountered in buds. Harris et al. (2006) [14] reported that leaves of naturally cold acclimated *R*. *catawbiense* plants resumed photosynthetic functions within a day of their transfer from wintery-cold outdoors to warmer growth chambers. This suggests that these leaves lack true endodormancy but undergo eco-dormancy. Overwintering leaves of rhododendrons also allow the measurement of freezing ‘tolerance’ (as opposed to ‘avoidance’) using lab-based freeze-thaw assay since leaf FT is conferred without supercooling [15].

We have investigated the physiological, molecular or genetic basis of cold acclimation in *Rhododendron* using various cultivars [16], species [13, 17], and F_2_ segregants [18–20] varying in their cold acclimation ability. Our Expressed Sequence Tag (EST)-based study with a selected form of native ‘hardy’ species (*R*. *catawbiense* ‘Catalgla’) revealed several candidate genes important for freezing tolerance [21–22]. Exposure to, and protection from, light stress by green leaves is also an important component of freezing tolerance in overwintering broadleaf evergreens. In this regard, cold-induced leaf curling/bending, i.e. thermonasty, in rhododendrons has been hypothesized to be an evolutionary adaptation to confer photoprotection [23–24]. Our previous work indicates that *Rhododendron* species contrasting in thermonastic behavior (presence or absence) also exhibit differential gene-expression responses along with distinct anatomy and photochemistry [25–27].

While transcript profiles provide valuable information, the data often does not clearly correlate with protein profiles [28–29]. Moreover, protein changes (function, transport and activation) are ‘downstream’ events relative to mRNAs and directly mediate cellular metabolisms [30]. In the present study, we characterized the proteome of leaves of *R*. *catawbiense*, a thermonastic species [31] collected at two time points: summer (non-acclimated) and winter (cold-acclimated; CA). For comparative proteomics, we also collected leaves in the winter from a non-thermonastic and lesser freezing tolerant species, *R*. *ponticum* [26]. Differentially abundant proteins were analyzed by two-dimensional differential gel electrophoresis (2-D DIGE) followed by mass spectrometry (MS), and several hypotheses were tested including: (1) that changes in the proteome could be detected associated with seasonal development of cold hardiness from the comparisons of non-acclimated and cold-acclimated *R*. *catawbiense* samples; and (2) that genotypic differences could be detected that are associated with different freezing tolerance and thermonastic abilities of the two species from the comparisons of cold-acclimated *R*. *catawbiense* and cold-acclimated *R*. *ponticum* samples.

## Results and discussion

### Overall changes in proteome during cold acclimation and quality of the data

To analyze the proteome response of rhododendron leaves during cold acclimation, proteins were extracted from leaves of non-acclimated (NA; Aug) and cold-acclimated (CA; Dec) plants of the freezing tolerant, thermonastic *R*. *catawbiense* ‘Catalgla’ (Cata.) and from CA plants of the less tolerant, non-thermonastic *R*. *ponticum* (Pont.). S1 Fig shows a flow chart of the experimental design. Leaf FT (LT_50_) of Cata.NA and Pont.NA plants was ~ -7°C and ~ -5°C, and ~ -34°C and -16°C for Cata.CA and Pont.CA samples, respectively, as assessed using a temperature-controlled freeze-thaw protocol coupled with ion-leakage measurements [25–26]. The three samples (Cata.NA, Cata.CA, and Pont.CA) were labeled with different CyDyes and fractionated simultaneously by 2D-DIGE. Because only three samples could be run at a time, a Pont.NA sample was not included in this study. Our experimental approach allowed us to first 1) characterize proteome changes associated with the seasonal development of FT, i.e. NA *versus* CA in Cata., and then 2) compare and contrast the CA proteomes of two *Rhododendron* species (Cata. and Pont.) varying in leaf FT and thermonastic behavior.

**Fig 1:**
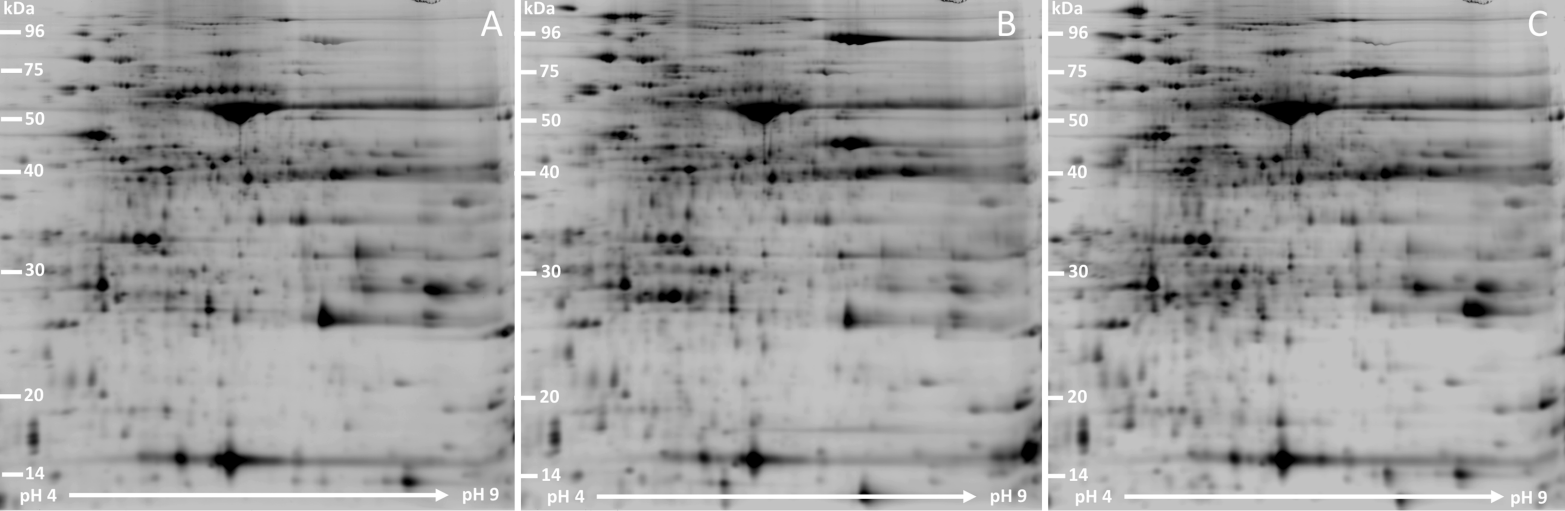
Representative gels of the experiment. 2DE gel analyses of proteins extracted from leaves. **(A)** non-acclimated *R*. *catawbiense*, Cata.NA. **(B)** acclimated *R*. *catawbiense*, Cata.CA. **(C)** acclimated *R*. *ponticum*, Pont.CA.

Cold acclimation induced significant changes in the proteome of *Rhododendron* leaves (Fig 1). Matching of protein spots across triplicate gels allowed calculation of relative protein abundance ratios of differentially accumulated proteins (fold changes) related to the other samples run at the same time. A number of reproducibly differentially abundant proteins were identified. We performed exploratory data analysis to visualize and summarize the overall quality of the data and identify general patterns (S2 Fig). A multi-dimensional scaling plot resulted in the clear separation of the protein data from the three possible pairwise comparisons (Cata.CA—Cata.NA, Pont.CA—Cata.NA, Pont.CA—Cata.CA) and grouping of protein data from the three replicates of the same pairwise comparisons. Comparative analysis showed larger differences between biological conditions than between replicates, indicating the reliability of experimental design and methodologies.

**Fig 2:**
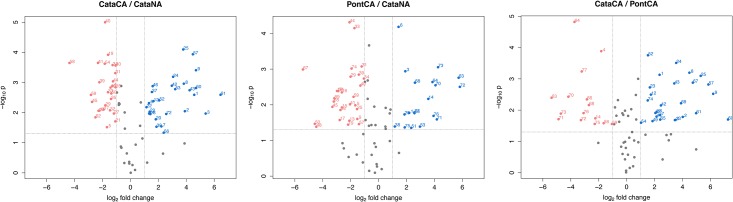
Volcano plot of significance against effect. Each dot represents one of the reproducible protein spots, with the –log_10_ of the *P* value plotted against the abundance difference between two biological conditions (log_2_ on the abscissa). Blue color denotes increased protein levels; red color denotes decreased protein levels; grey color denotes spots in meaningful sectors (effect factor <|2-fold|, *P* >0.05, or both).

**Fig 3:**
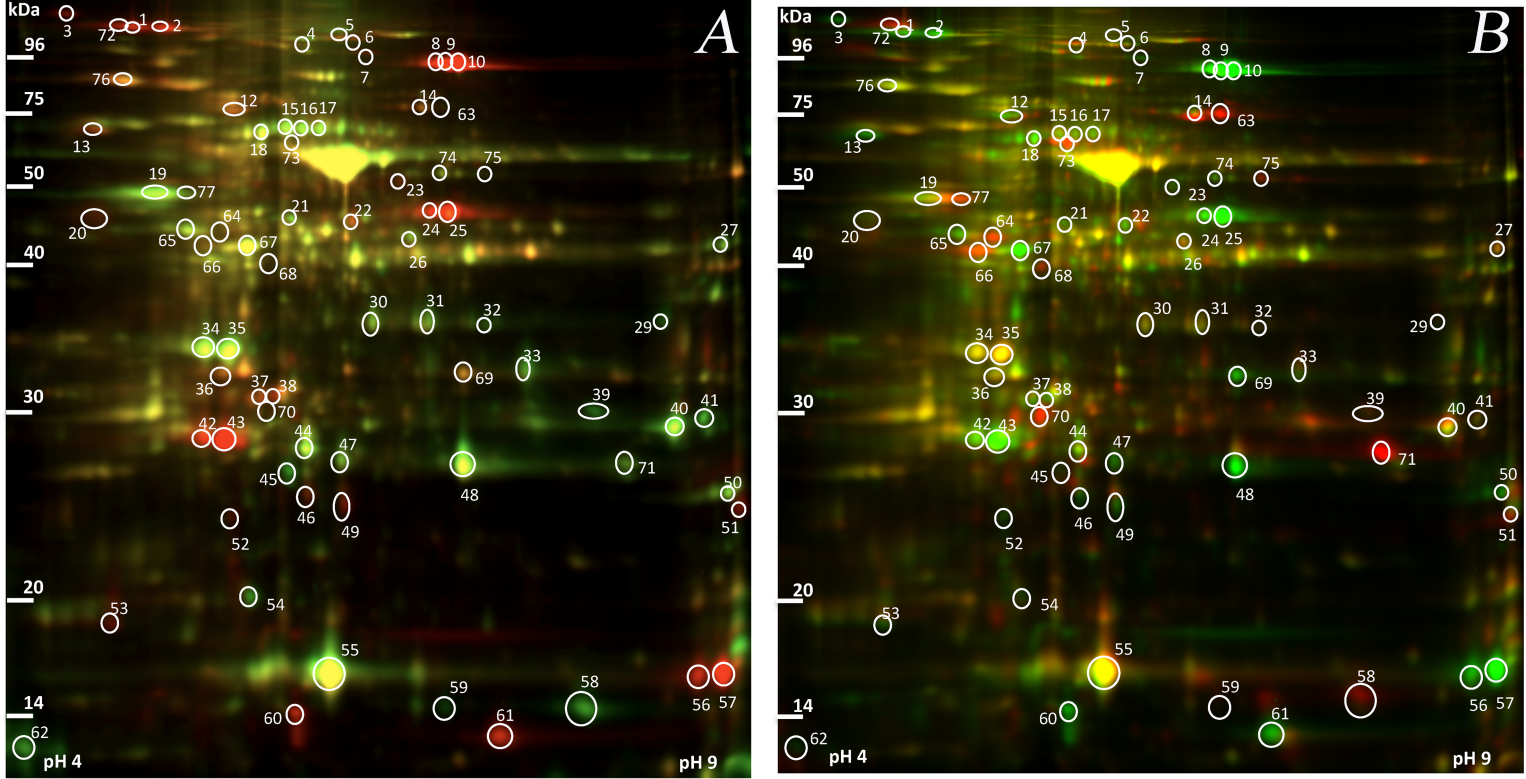
2D DIGE analyses of leaf proteins. **(A)** Protein extracts from acclimated *R*. *catawbiense* samples (Cata.CA; labeled with red) and non-acclimated *R*. *catawbiense* samples (Cata.NA; labeled with green). **(B)** Protein extracts from *R*. *catawbiense* samples (Cata.CA; labeled with green) and *R*. *ponticum* samples (Pont.CA; labeled with red). The positions of 72 differentially abundant protein spots are indicated.

To identify proteins of interest, we used the following criteria: spots with abundance ratios ≥ |2-fold| and significant differences (*t*-test *P* < 0.05) between two biological conditions (based on the three replicates). Fig 2 shows a plot of the *P* values obtained against the magnitude of the effect. It illustrates the statistical significance of the differences (*P* values) as opposed to cut-offs strictly based on fold changes. The two vertical reference lines indicate a two-fold cut-off for either an increase or decrease in abundance, whereas the horizontal line represents the test-wise threshold of *P* = 0.05 dividing the plot into meaningful sectors. Only those spots in the upper left and right sectors have high significances (*P<*0.05) and high-fold changes (>|2|). A comparison among conditions revealed that 72 spots had significantly different abundances in at least one comparison. A representative gel is shown in Fig 3.

**Fig 4:**
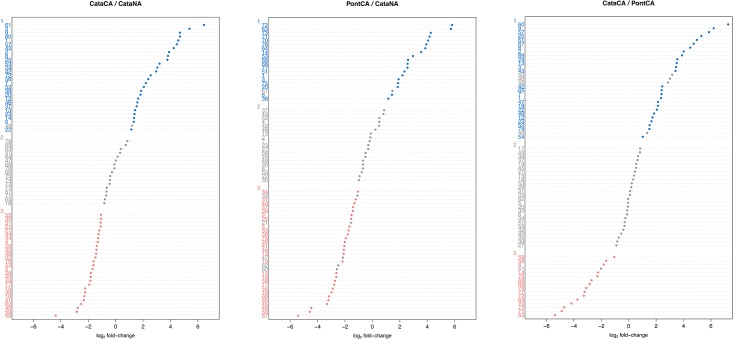
Spot abundance profiles. Different patterns of protein induction (blue) or suppression (red) are shown for each pair-wise comparison. Grey color denotes non-significant regulation (effect factor <|2-fold|, *P* >0.05, or both).

The spot-volume profiles of the 72 spots showed different abundance patterns (Fig 4). Concerning the pairwise comparison Cata.CA-Cata.NA, we identified a total of 54 differentially abundant protein spots, which included 27 increasing and 27 decreasing in abundance. The log_2_ ratios of the changes ranged from a 4.37-fold decrease to a 6.52-fold increase in abundance. Next, 47 spots showed differential accumulation in Pont.CA relative to Cata.NA plants (19 higher in abundance, 28 lower in abundance), with log_2_ ratios ranging from a 5.44-fold decrease to a 5.89-fold increase. Finally, 41 spots were found to accumulate differently in Cata.CA plants compared to Pont.CA plants (26 higher in abundance, 15 lower in abundance) with log_2_ ratios ranging from a 7.83-fold decrease to a 5.45-fold increase.

Protein profiles differed not only between NA and CA plants of Cata., but also between CA plants of both genotypes. Pont.CA plants did not show an increase in abundance of as many spots as Cata.CA did relative to Cata.NA (19 *versus* 27). Also, more spots (26) were higher in abundance in Cata.CA plants *versus* 19 spots that were lower in abundance in Cata.CA compared to Pont.CA.

From the 72 differential spots, we sorted those with the largest differences between Cata.CA—Cata.NA and Pont.CA—Cata.CA comparisons, and chose 56 spots to excise and submit for MS-based identification. Statistical data and protein identification data for those spots are presented in S1 Table. Gene ontologies (GO) were assigned to proteins based on Blast2GO annotation. As a result, the 56 spots could be classified into one or more ontologies: 49 were classified according to biological processes, 47 by molecular functions, and 32 were classified in terms of cellular components; 30 were classified in all three ontologies. These GO terms and ontologies were the basis for further sequence classification into functional groups.

To best organize our findings, we first discuss the spots changing in abundance during seasonal cold acclimation in Cata. This is found under the ‘seasonally-dependent regulation’ section below. About half of these spots (24) also accumulated differently between CA plants of the two genotypes. Their potential functions are further discussed in the section ‘genotype-dependent regulation’. Spots whose levels did not change during cold acclimation in Cata. but did show different levels between CA Cata. and CA Pont. plants are also discussed in the second part of this section.

### Seasonally-dependent regulation

#### Up-regulated proteins

To understand the functions of the 27 protein spots increasing in abundance during cold acclimation of the freezing tolerant genotype ‘Catalgla’, the spots were classified into 9 functional categories including energy and carbohydrate metabolism (8 spots), regulation/signaling (6 spots), response to stress (5 spots), secondary metabolism (2 spots), protein metabolism (2 spots), and structural components, transport, miscellanea and unknown function categories with 1 spot each (S3 Fig).

The spots showing the largest increase in abundance were in the stress response category (up to 6.45 log_2_ ratios). These spots represented 18.5% of the up-regulated proteins in the genotype ‘Catalgla’, and included a dehydrin, a cold shock domain protein and several monodehydroascorbate reductase (MDAR) variants. The largest number of spots increasing in abundance fell in the energy production and carbohydrate metabolism category (~30% of the spots), with log_2_ ratios ranging from 1.42 to 3.20 in response to cold acclimation. Proteins from this group are involved in the Calvin cycle, tricarboxylic acid (TCA) cycle, glycolysis and the pentose phosphate pathway. The regulation/signaling group (log_2_ ratios of 2.05 to 4.69) contained 4 of the 10 spots with the highest increases in abundance, suggesting that transcriptional and translational regulation are major processes during cold acclimation. Moreover, two protein spots involved in secondary metabolism (phenylpropanoid biosynthesis and carotenoid metabolism), two involved in protein degradation metabolism and one outer membrane protein involved in metabolite transport and maintenance of membrane permeability (temperature-induced lipocalin) increased in abundance during cold acclimation. The set of proteins increasing in abundance also included an unknown protein (Table 1).

**Table 1:** Spots up-regulated in *R*. *catawbiense* during cold acclimation and their log_2_ abundance ratios related to non-acclimated *R*. *catawbiense* (CataNA) or acclimated *R*. *ponticum* plants (PontCA). Abundance ratios between PontCA/CataNA for those spots are also shown. Table shows only significant data.

Spot Id.	Annotation	Biological function/Pathway	CataNA	PontCA	PontCA/CataNA
	**Energy and Carbohydrate metabolism**				
Spot 6	ribulose biphosphate carboxylase oxygenase large subunit	Photosynthesis. Calvin cycle	1.33		1.43
Spot 7	ribulose bisphosphate carboxylase oxygenase large subunit	Photosynthesis. Calvin cycle	2.21	2.40	
Spot 12	bisphosphoglycerate independent phosphoglycerate mutase	Glycolysis	1.64	1.74	
Spot 13	ATPase subunit 4	Mitochondrial electron transport / ATP synthesis	1.42	3.50	-2.09
Spot 14	NADP-dependent malic enzyme-like	TCA cycle /malate metabolic process/pyruvate metabolism/photosynthesis	1.34	-2.24	3.56
Spot 20	ribulose bisphosphate carboxylase oxygenase chloroplastic	Photosynthesis. Calvin cycle	1.81		1.85
Spot 53	chlorophyll binding protein	Photosystem II	3.20		2.96
Spot 56	ribulose bisphosphate carboxylase small chain	Photosynthesis. Calvin cycle	2.38		
	**Regulation / Signaling**				
Spot 1	DNA-directed RNA polymerase subunit ß	RNA transcription	4.57	2.36	2.20
Spot 8	elongation factor 1-alpha	Translation regulation. Protein synthesis	3.82	4.49	
Spot25	pentatricopeptide repeat-containing mitochondrial protein	Translation regulation	3.78	5.30	-1.54
Spot 42	RNA polymerase beta subunit	RNA transcription	2.96	2.45	
Spot 52	BTB/POZ domain containing protein	Protein homooligomerization	2.05	1.53	
Spot 60	DEAD-box ATP-dependent RNA helicase	RNA metabolism	4.69	7.26	
Spot 72	pentatricopeptide repeat-containing protein	Translation regulation	2.56	-3.27	5.82
	**Stress-related proteins / defense**				
Spot 3	dehydrin erd10-like	Response to stress	5.38	3.46	1.91
Spot 22	monodehydroascorbate reductase	Redox	1.14	2.06	
Spot 23	monodehydroascorbate chloroplastic	Redox	1.38	1.66	
Spot 61	cold shock domain protein 3	Response to stress. Chaperone	6.45	4.98	1.46
	**Secondary metabolism**				
Spot 2	4-Coumarate:CoA ligase	Phenylpropanoid biosynthesis	3.89	4.02	
Spot 43	carotenoid cleavage dioxygenase	Isoprenoid / Carotenoid metabolism	4.23	3.41	
	**Protein metabolism**				
Spot 37	NAD(P)-binding rossmann-fold	Protein degradation	1.53		
Spot 38	NAD(P)-binding rossmann-fold	Protein degradation	1.86		1.16
	**Structural components**				
Spot 57	cellulose synthase-like 6	Cell wall	4.47	5.86	-1.41
	**Transport**				
Spot 46	temperature-induced lipocalin (outer membrane lipoprotein Blc-like)	Transport	1.59		
	**Unknown**				
Spot 9	unknown protein		4.69	6.20	-1.52
Spot 24	hypothetical protein PHAVU_011G166900g		3.05	3.55	

#### Down-regulated proteins

Twenty-seven spots decreased in abundance in CA leaves of the genotype ‘Catalgla’ as well. MS data was obtained for 16 of the most down-regulated spots, which were grouped into two functional categories: energy and carbohydrate metabolism (14 spots) and stress response-related proteins (2 spots; S3 Fig). Half of the spots within the ‘energy and carbohydrate metabolism’ category were part of the group of 10 spots that decreased the most in abundance. These protein spots are involved in photosynthesis, the TCA cycle and oxidation-reduction processes. Two more spots (abscisic stress ripening protein and a superoxide dismutase) in the stress-related protein category also decreased sharply during cold acclimation (Table 2).

**Table 2:** Spots down-regulated in *R*. *catawbiense* during cold acclimation and their log_2_ abundance ratios related to non-acclimated *R*. *catawbiense* (CataNA) or acclimated *R*. *ponticum* plants (PontCA). Abundance ratios between PontCA/CataNA for those spots are also shown. Table shows only significant data.

Spot Id.	Annotation	Biological function/Pathway	CataNA	PontCA	PontCA/CataNA
	**Energy and Carbohydrate metabolism**				
Spot 5	ribulose biphosphate carboxylase oxygenase large partial	Photosynthesis. Calvin cycle	-1.69		-1.66
Spot 16	ribulose biphosphate carboxylase oxygenase large partial	Photosynthesis. Calvin cycle	-2.29		-2.63
Spot 19	ribulose bisphosphate carboxylase oxygenase chloroplastic isoform	Photosynthesis. Calvin cycle	-1.61		-2.18
Spot 29	carbonic anhydrase	TCA.Nitrogen metabolism	-1.80		-2.01
Spot 32	ferredoxin NADP leaf chloroplastic	Photosynthesis, light reaction	-1.47		
Spot 35	oxygen-evolving enhancer protein chloroplastic	Photosystem II. Photoinhibition	-1.40		
Spot 39	carbonic anhydrases	TCA. Nitrogen metabolism	-2.23		-1.41
Spot 40	carbonic anhydrases	TCA. Nitrogen metabolism	-1.82		-1.30
Spot 41	carbonic anhydrases	TCA. Nitrogen metabolism	-2.33		-2.05
Spot 48	oxygen evolving complex	Photosystem II	-1.43		-4.46
Spot 55	ribulose bisphosphate carboxylase small chain	Reductive pentose-phosphate cycle. Photorespiration.	-1.07		
Spot 58	ribulose bisphosphate carboxylase small chain	Reductive pentose-phosphate cycle. Photorespiration.	-4.37	-1.65	-2.73
Spot 59	ribulose bisphosphate carboxylase small chain chloroplastic	Photorespiration. Carbon fixation.	-2.83	-1.79	-1.79
Spot 62	plastocyanin chloroplast	Oxidation–reduction process	-2.51		
	**Stress-related proteins / defense**				
Spot 45	abscisic stress ripening	Response to stress	-2.75		-3.30
Spot 54	superoxide dismutase	Response to oxidative stress	-1.84	1.03	-2.88

### Genotype-dependent regulation

Next, we aimed to identify differential changes in protein levels related to the different freezing tolerance levels and / or thermonastic responses of the two genotypes. Of the spots which changed in abundance during seasonal cold acclimation of Cata., 24 accumulated to different levels in Cata.CA and Pont.CA plants. From this set, 19 spots had higher levels in Cata.CA and 5 spots had higher levels in Pont.CA. Their potential biological functions are discussed in the following sections.

#### Stress-related proteins

A group of stress-related proteins showing the largest increase in abundance in Cata. also showed, in general, the largest differences compared to Pont. plants. This group is comprised of a cold shock domain protein 3 (CSDP), a dehydrin erd10-like protein, and two monodehydroascorbate reductases (MDAR). The CSDP level was ~5-fold higher in Cata.CA than in Pont.CA. Annotation of its sequences found cellular components associated with nuclear and cytoplasmic compartments, suggesting a role in multiple complexes. The presence of an S1-like motif in the sequence highlights a function as a binding-protein. A search of the Plant Transcription Factor Database (PlantTFDB v3.0; http://planttfdb.cbi.pku.edu.cn/) suggests a potential C3H-type zinc finger, a TF family that is associated with important roles in responses to cold stress [32–33]. The accumulation of CSDP3 regulates FT in *Arabidopsis* independently of the CBF/DREB1 pathway [34] and a nucleic acid-binding cold-shock protein, WCSP1, accumulates specifically during cold acclimation of wheat. [35]. CSDPs could confer cold tolerance by functioning as RNA chaperones [35–36]. Since freezing-temperatures are associated with an enhanced risk of RNA misfolding, increasing the abundance of RNA chaperones is a molecular strategy that may protect against freeze-induced damage by ensuring maintenance of the RNAs biological functions.

Cata.CA leaves also accumulated a dehydrin at ~3.5-fold higher abundance than did Pont.CA (spot #3, Table 1). The broad implication of dehydrins in protecting against freeze damage is well documented in the literature by their high accumulation in both herbaceous [37–39] and woody plants [40–44]. They play multiple roles in FT including cryoprotection, stabilization of cell membranes and protein denaturation prevention [45].

Finally, the accumulation of two MDAR variants (spots #22, #23) was ~ 1.7 to 2.1-fold higher in Cata. than in Pont. (Table 1). MDAR is an enzymatic component of the ascorbate-glutathione cycle, one of the major antioxidant pathways in plants, and is associated with the chloroplast stroma. Maintenance of photostasis in winters can be problematic for broad-leaved evergreens, such as rhododendrons, because utilization of light energy through photosynthesis is significantly reduced while light harvesting is not. This excess energy, if not efficiently dissipated as heat, can result in ‘ROS overload’. We hypothesize that upregulation of MDARs is one of the strategies to detoxify ROS in CA leaves of Cata. and Pont. This hypothesis is consistent with other reports in the literature [46–47]. Moreover, increased activities of several antioxidant enzymes (ascorbate peroxidase, catalase, superoxide dismutase) during seasonal cold acclimation have been observed in our laboratory for these two *Rhododendron* species [26]. This seems to support our earlier observations [25] that Cata. leaves in winter are more photosensitive than Pont. leaves based on their chlorophyll fluorescence response, and thus may require more robust antioxidant machinery.

#### Regulation, signaling and protein metabolism

A number of spots encoding proteins involved in regulation, signaling, and protein metabolism were more abundant in Cata.CA than in Pont.CA. These included RNA polymerase (spot #1, #42), elongation factor 1-alpha (spot #8), a pentatricopeptide repeat-containing protein (spot #25), and a protein containing a BTB/POZ domain (spot #52), a protein-protein interaction module involved in regulation of gene expression through the local control of chromatin conformation [48]. Interestingly, a spot with homology to a DEAD box RNA helicase was the spot with the largest difference in abundance between the two genotypes (spot #60, log_2_ ratio >7.2 higher level in Cata.). It is also one of the five most up-regulated proteins during cold acclimation in Cata. RNA helicases are involved in every step of RNA metabolism, and some of them are essential for freezing tolerance [49–50]. Although the specific role of this RNA helicase remains to be established, it may be a good candidate for a new freezing adaptation marker in rhododendron.

#### Energy and carbohydrate metabolism

Levels of proteins linked to energy and carbohydrate metabolism increased during cold acclimation and many were higher in Cata.CA than in Pont.CA. The higher levels observed for RuBisCO large subunit (spot #7), 2,3-bisphosphoglycerate-independent phosphoglycerate mutase (spot #12), and ATPase subunit 4 (spot #13) suggest higher formation of storage carbohydrates and upregulation of carbohydrate catabolism in Cata. *versus* Pont. including glycolysis and mitochondrial electron transport, which is consistent with an enhanced energy demand during active cold acclimation [51]. Still, photosynthesis and carbohydrate metabolism are the most down-regulated processes that we observed during cold acclimation in Cata., denoting important energy and photosynthesis adjustments (Table 2). This is consistent with the reduced photosynthetic rates and reduced levels of various photosynthesis-related cDNAs observed during cold acclimation in the species [26, 52]. The complexity of the regulation of photosynthesis-related proteins is illustrated by the increases and decreases in abundance of the different subunits of RuBisCO. The small subunit of RuBisCO (spots #58, #59) showed lower levels in Cata. than in Pont.; this may be associated with the differential photosynthetic behavior (light response curves) of the two species. While both species exhibit reduced photosynthetic capacity from summer to fall, Pont. has higher photosynthesis and is less photoinhibited [26]. The higher level of RuBisCO activase in Pont. (S2 Table) supports this observation.

The winter reduction in photosynthetic capacity by evergreens can also be accomplished by reduced leaf chlorophyll [26], as well as the degradation of oxygen-evolving complex (OEC) and other PSII core proteins [53]. In this study we observed a significant reduction in OEC protein levels during cold acclimation of Cata. (spot #35 and #48; Table 2). Zarter et al. (2006) [53] suggested that down-regulation or degradation of OEC (and water-splitting sites and PSII core) removes the source of electrons for photochemistry and prevents transfer of electrons to oxygen and formation of destructive superoxide. It is, therefore, an adaptive response in CA winter leaves of evergreens. A relatively lower abundance of OECs in Cata. leaves compared to Pont. may be associated with the greater photo-sensitivity of the former, as suggested by Wang et al., (2008; 2009) [25–26].

Our results also indicate a strong upregulation of chlorophyll a/b binding (CAB) proteins in CA leaves of the two species compared to Cat. NA (spot #53, Table 1 for both species; and spot #70, Supp. Table 1 for Pont.) To maintain photostasis during winter, evergreens reduce their light-harvesting capacity (reduce chlorophyll antenna size), down-regulate OEC, and/or upregulate antioxidant machinery (discussed above), besides employing mechanisms for non-photochemical dissipation of excess absorbed solar energy [54]. Adams and Adams (2006) [55] suggest that such energy dissipation in the form of heat involves the de-epoxidation state of xanthophyll cycle pigments as well as CABs, hence the upregulation of the latter. Our earlier work and that of others have reported a substantial upregulation of specific CABs (i.e. Elips, early light induced proteins), in winter leaves of rhododendrons [17, 52], and Elips and PsbS proteins in several conifer species [53]. In this study we could not assign specific identity to CAB members. However, notably one of the CABs accumulated at a higher level in Pont.CA than in Cata.CA (spot #70; Supp. Table 2; log_2_ Cata.CA/Pont.CA = -4.70). This differential response by the two species may be associated with their differential thermonasty behavior. If the Pont. species cannot curl its leaves to avoid absorbing excess solar energy, it may respond with higher levels of CAB proteins.

#### Secondary metabolism and structural components

Cold acclimation also resulted in a higher abundance of enzymes involved in secondary metabolism and in cell wall formation. One of the enzymes that was induced during cold acclimation in rhododendron and was more abundant in Cata.CA than Pont.CA was 4-coumarate: CoA ligase (4CL; spot #2), which is involved in phenylpropanoid metabolism. This pathway produces phenolic compounds as precursors of suberins and lignins [56]. Lignins are a major component of the cell wall, and the reinforcement of plant cell wall through lignin biosynthesis is a general plant response to abiotic stress [51, 57]. Another enzyme involved in cell wall formation, cellulose synthase (spot #57), was induced with cold acclimation in Cata. and was more than 5.8-fold more abundant in Cata.CA than in Pont.CA. Alterations in these two enzymes may indicate substantial cell wall remodeling and increased lignification as a response to freezing, providing a barrier against dehydration stress [51].

We also observed higher levels of another enzyme involved in secondary metabolism (spot #43) with cold acclimation, and higher levels were found in Cata.CA than in Pont.CA. The annotation of the spot confirmed a 9-cis-epoxycarotenoid dioxygenase (NCED), which catalyzes the primary steps of ABA biosynthesis in plastids [58, 59]. This finding might suggest that cold-induced FT in rhododendron involves an ABA-dependent regulatory pathway.

#### Unknown proteins

Although we attempted several times to identify spot #9, we could not match it against any other sequences in the database with even low confidence. Spot #9 is the third most up-regulated protein in Cata.CA and, after spot #60, shows the biggest difference in levels between the CA genotypes (log_2_ ratio >7.2 higher level in Cata. *versus* Pont.). This pattern of regulation seems to suggest a protein of major importance in the acquisition of FT in Cata. It would be of interest to further characterize it and establish its role in FT.

#### Constitutive abundance levels

Finally, we identified a set of 11 spots non-seasonally-regulated in Cata. during cold acclimation but displaying different abundances compared to Pont. (S2 Table). Three of these spots (#49, #67, #69) were more abundant in either Cata.NA or Cata.CA than in Pont.CA. The first, glutathione peroxidase (spot #49) protects cell membranes during freezing by removing lipid peroxides [60]. The second is glutamine synthetase (spot #67), and glutamate availability also is associated with FT [39, 45]. The third is a plastid-lipid-associated protein (spot #69), an abiotic stress responsive protein with a role in protecting thylakoid membranes during freezing [61, 62]. The fact that these three proteins are associated with FT, but are not regulated during cold acclimation in Cata., suggests that higher activity, or more abundant levels constitutively of certain protective proteins, may also contribute to higher FT of Cata. than Pont.

In conclusion, this is a first report to our knowledge on discerning the proteomics of overwintering leaves with substantial freeze-tolerance. Moreover, since thermonasty in overwintering broad-leaved rhododendrons has been hypothesized to be a photoprotective strategy, the comparative analysis of a thermonastic *versus* non-thermonastic *Rhododendron* species in this study allowed the deciphering of putative photoprotection-related proteome differences associated with cold-acclimation.

## Materials and methods

### Plant material

Plants from two *Rhododendron* species [*R*. *catawbiense* Michx. ‘Catalgla’ (Cata.) and *R*. *ponticum* L. ‘RSBG 76/411’ (Pont.); RSBG is Rhododendron Species Foundation and Botanical Garden] were vegetatively propagated (semi–hardwood cuttings) and grown in 19–L plastic pots at the David G. Leach Research Station of Holden Arboretum (Kirtland, OH, USA). About 2–year–old containerized plants were brought to the Department of Horticulture at Iowa State University (ISU) in Ames, IA, USA and maintained for research purposes. All plants were grown in Fafard mix 52 (Conrad Fafard, Inc., Agawam, MA, USA) [pine bark (60%); peat, perlite and vermiculite (40%)] outside the ISU Horticulture greenhouse (latitude 42° 1' N, longitude 93° 38' W). The granular slow-release fertilizer (5% N, 2% P, 2% K, 2% Ca, 2% S, and 2% Fe; Suståne / Natural Fertilizer of America, Inc., Cannon Falls, MN, USA) was applied to the medium surface every three weeks during the growing season from May to August. Plants were watered as needed throughout the experiment. Four year-old plants were used for this study. Plants were maintained outdoors and acclimated naturally from August to December. Fully expanded leaves from the current year growth (2 leaves from each of the 4 plants per species) were collected on sunny mornings (~ 09:00 h) for each protein extraction sampling—non-acclimated (NA) samples in mid-August and cold acclimated (CA) in late December—and frozen at –80°C to be used for protein extractions.

#### Protein extraction, CyDye labeling, and 2D-DIGE

Proteins were extracted with TCA-phenol [63] from Cata.NA, Cata.CA, and Pont.CA samples and quantified according to Esen (1978) [64]. Two-dimensional differential in-gel electrophoresis (2D-DIGE) was performed at Applied Biomics, Inc. (Hayward, CA) as described previously [44]. Briefly, protein extracts from rhododendron leaves were denatured by adding an equal volume of lysis buffer containing 7M urea, 2M thiourea, 4% 3-((3-cholamidopropyl) dimethyl ammonio)-1-propanesulfonate (CHAPS), followed by addition of 30 mM Tris-HCl, pH 8.8. For each set of three samples to be run on a single gel (Cata.NA, Cata.CA, and Pont.CA), 30 μg of sample protein were then labeled with 1.0 μl CyDye dilution of Cy2, Cy3, or Cy5 (1:5 dilution in a 1 nmol/μl DMF stock; Amersham Biosciences, Piscataway, NJ). CyDye labeling was stopped by adding 0.7 μl of 10 mM L-lysine and incubating at 4°C for 15 min. Equal amounts of the three labeled samples were combined together, along with an equal volume of 2X 2-D sample buffer (8 M urea, 4% CHAPS, 20 mg/ml dithiotreitol (DTT), 2% pharmalytes and a trace amount of bromophenol blue) and 100 μl of destreak solution (GE Healthcare Biosciences, Pittsburgh, PA). Sample volumes were adjusted to 260 μl total by adding rehydration buffer (7 M urea, 2 M thiourea, 4% CHAPS, 20 mg/ml DTT, 1% pharmalytes and trace amount of bromophenol blue). Each set of three labeled samples were subjected simultaneously to isoelectric focusing (IEF) on a 13-cm precast non-linear immobilized pH gradient strip (pH 4–9, Amersham Biosciences). We have used this pH range to identify succesfully a set of proteins induced during the cold acclimation progression in blueberry [33].Next, the samples were separated in the second dimension based on size by sodium dodecyl sulfate polyacrylamide gel electrophoresis (SDS-PAGE).

### Image scan and data analysis

Gels were run in triplicate, scanned, and analyzed as described previously [44]. To summarize, scanning was performed using a Typhoon Trioscanner (Amersham BioSciences) following the manufacturer’s protocol. Scanned images were processed using Image Quant software (Amersham BioSciences, v.5.0). Protein abundance was assessed by differential in-gel analysis (DIA). The quantitative analysis of protein spots was performed using DeCyder software (Amersham Biosciences, v.6.5). Quantitative comparisons were calculated between samples run at the same time and pair-wise volume ratios were calculated for each protein spot and used to determine relative fold changes ratios. Fold changes were log_2_ transformed. Student’s *t*-test was performed using the log2 normalized average spot volume ratios for all spots detected from the three replicate experiments. Only statistically significant results (*P*<0.05), and differentially abundant proteins with a ratio > 2-fold (log2 of 1) difference in one condition (increase or decrease in abundance), were chosen for mass spectrometry.

### Protein identification by mass spectrometry

Based on 2D-DIGE and data analysis by DeCyder software, spots of interest were subjected to in-gel trypsin digestion, peptide extraction, desalting, and spotting on a MALDI plate followed by MALDI-TOF/TOF to determine protein identity as described in [44]. In brief, mass spectra (MS) of the peptides in each sample were obtained using Applied Biosystems Proteomics Analyzer (Foster City, CA). The 10–20 most abundant peptides in each sample were subjected to further fragmentation and tandem mass spectrometry (MS/MS) analysis. Combined MS and MS/MS spectra were submitted for database search using GPS Explorer software equipped with the MASCOT search engine to identify proteins from primary sequence databases (NCBI nr). The highest scoring hit for each spot was used as the protein identification label. Candidates with either protein score C.I.% or ion C.I.% greater than 95 were considered significant.

### Functional classification and bioinformatic tools

The Gene Ontology Functional Annotation Tool Blast2GO version 3.2 [65] was used to assign GO identities and enzyme commission numbers to proteins identified by MS. For the annotation, the following configuration settings were used: BLASTP against NCBI non-redundant (nr) protein database, *E*-value filter ≤10^−6^, length cutoff of 33, maximum 10 BLAST hits per sequence, and annotation cutoff of 50. Furthermore, to improve annotation, InterProScan was performed and results were merged to GO annotation. The program Blast2GO was also used to assign biological functions, cellular components, and cellular processes to the sequences. Protein spot ratios data and Blast2GO outputs were passed through a custom pipeline built with R to analyze data, build clusters and generate some figures. The base R package (R Core Team 2015) and the open-source interface RStudio (http://www.rstudio.com/) were used [66–67].

## Supporting information

S1 Fig Flowchart of the experimental design. (PDF)

S2 Fig MDS and clustering plot showing relationships between sample types. **(A)** Multidimensional scaling (MDS) plot. Distance between sample labels indicates similarity. **(B)** Cluster dendrogram. Number of branches separating samples indicates similarity.
(TIF)

S3 Fig Functional classification of cold-responsive proteins regulated in the freezing tolerant genotype ‘Catalgla’. (TIFF)

S1 Table Peptide summary identification by spot number, log2 fold changes and *P* values for different pair-wise comparisons. Only *P* values < 0.05 are shown.
(XLS)

S2 Table Spots non-seasonally regulated in *R*. *catawbiense* during cold acclimation with significant higher (log_2_ abundance ratio > 0) or lower levels (log_2_ abundance ratio < 0) related to acclimated *R*. *ponticum* plants (PontCA). (DOCX)
