# Supplementary material for: Proteome dynamics of cold-acclimating Rhododendron species contrasting in their freezing tolerance and thermonasty behavior
Source: PLoS One. 2017 May 23;12(5):e0177389. doi: 10.1371/journal.pone.0177389 (PMC5441609; doi:10.1371/journal.pone.0177389)
Supplement: S1 Fig — (PDF) [file pone.0177389.s001.pdf]

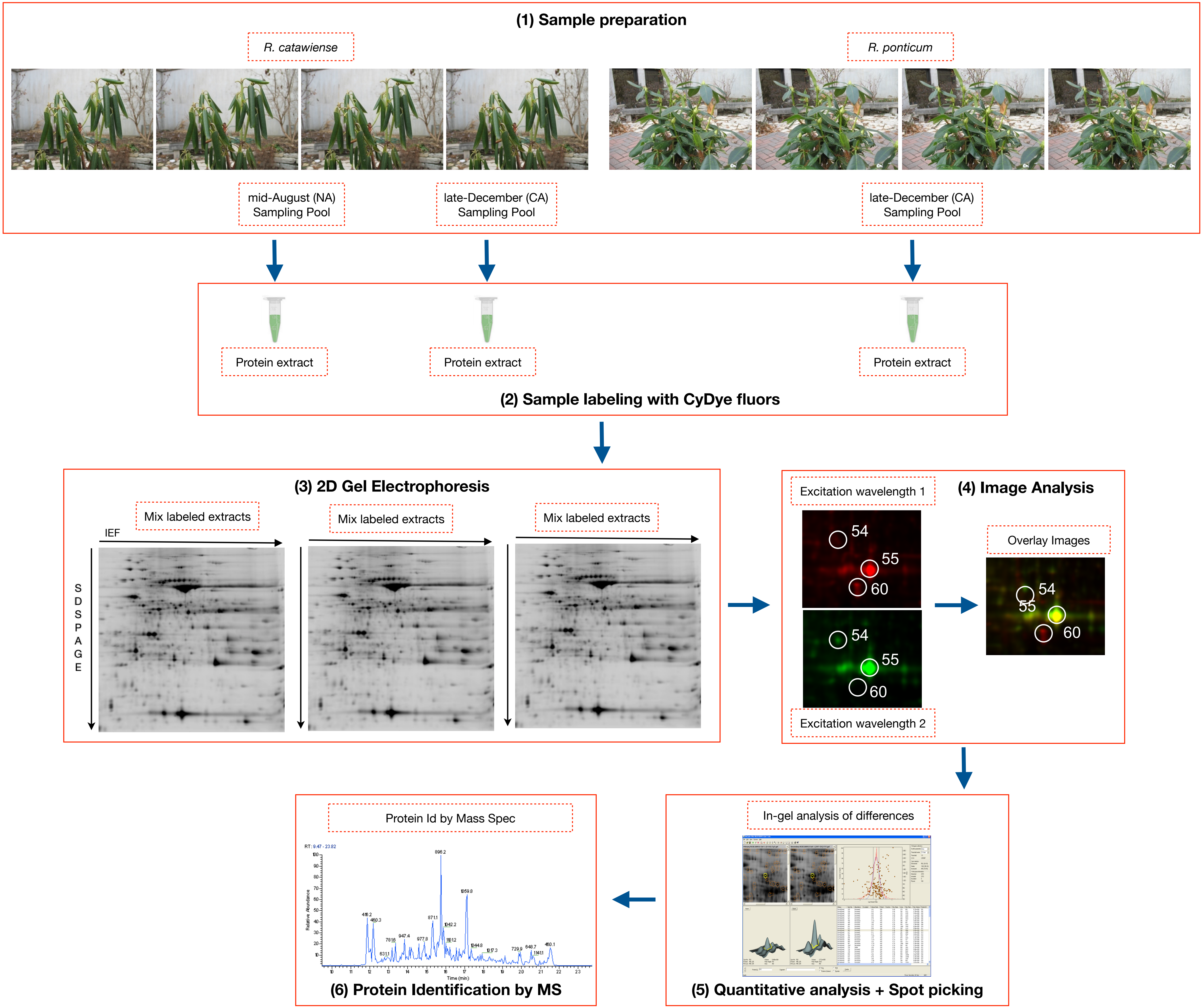

(1) Sample preparation
R. catawiense
R. ponticum
mid-August (NA) Sampling Pool
late-December (CA) Sampling Pool
late-December (CA) Sampling Pool
Protein extract
Protein extract
Protein extract
(2) Sample labeling with CyDye fluors
(3) 2D Gel Electrophoresis
Mix labeled extracts
Mix labeled extracts
Mix labeled extracts
IEF
SDSPAGE
(4) Image Analysis
Excitation wavelength 1
54
55
60
54
55
60
Excitation wavelength 2
Overlay Images
54
55
60
In-gel analysis of differences
(5) Quantitative analysis + Spot picking
Protein Id by Mass Spec
Relative Abundance
Time (min)
(6) Protein Identification by MS
