# Supplementary figures and images for: Proteome dynamics of cold-acclimating Rhododendron species contrasting in their freezing tolerance and thermonasty behavior
Source: PLoS One. 2017 May 23;12(5):e0177389. doi: 10.1371/journal.pone.0177389 (PMC5441609; doi:10.1371/journal.pone.0177389)

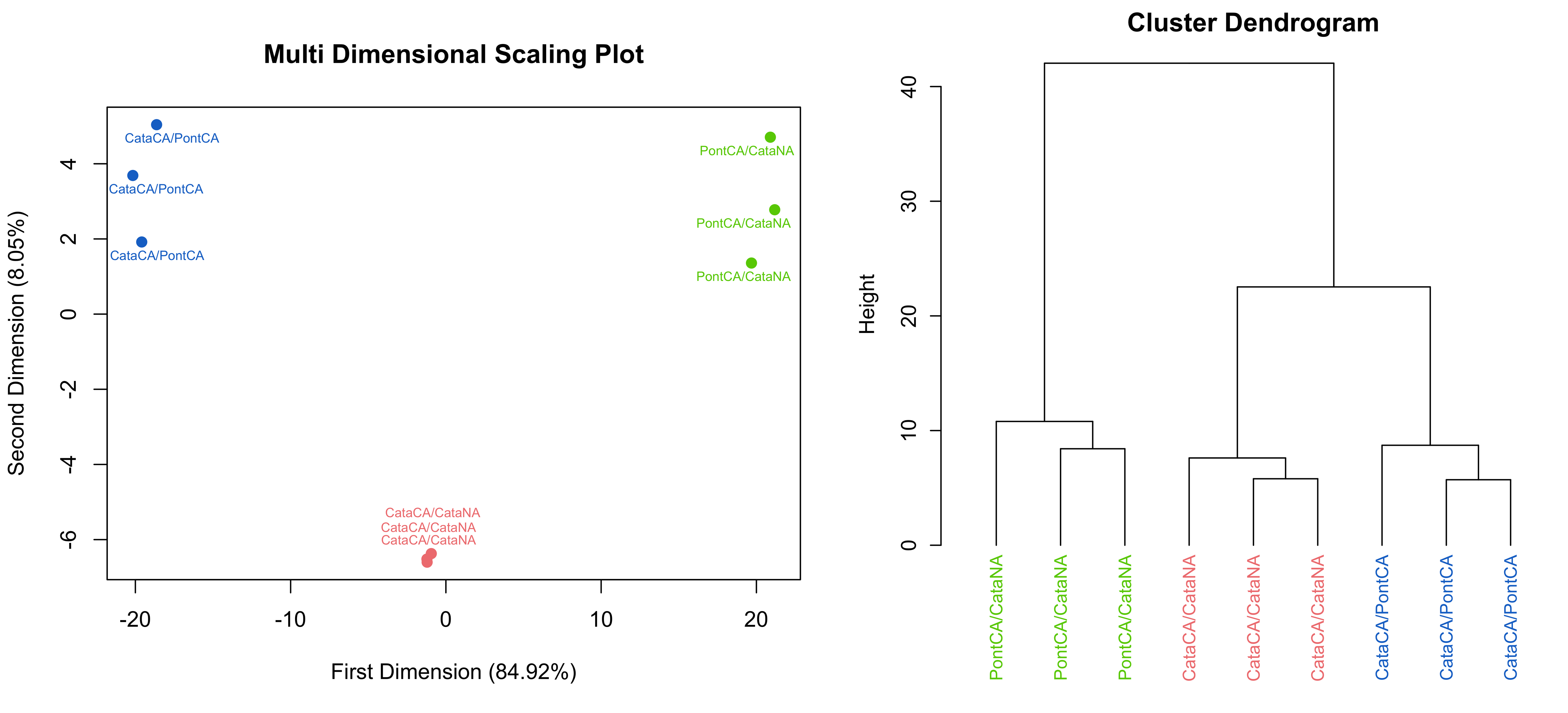

Supplement: S2 Fig — (A) Multidimensional scaling (MDS) plot. Distance between sample labels indicates similarity. (B) Cluster dendrogram. Number of branches separating samples indicates similarity. (TIF) [file pone.0177389.s002.tif]

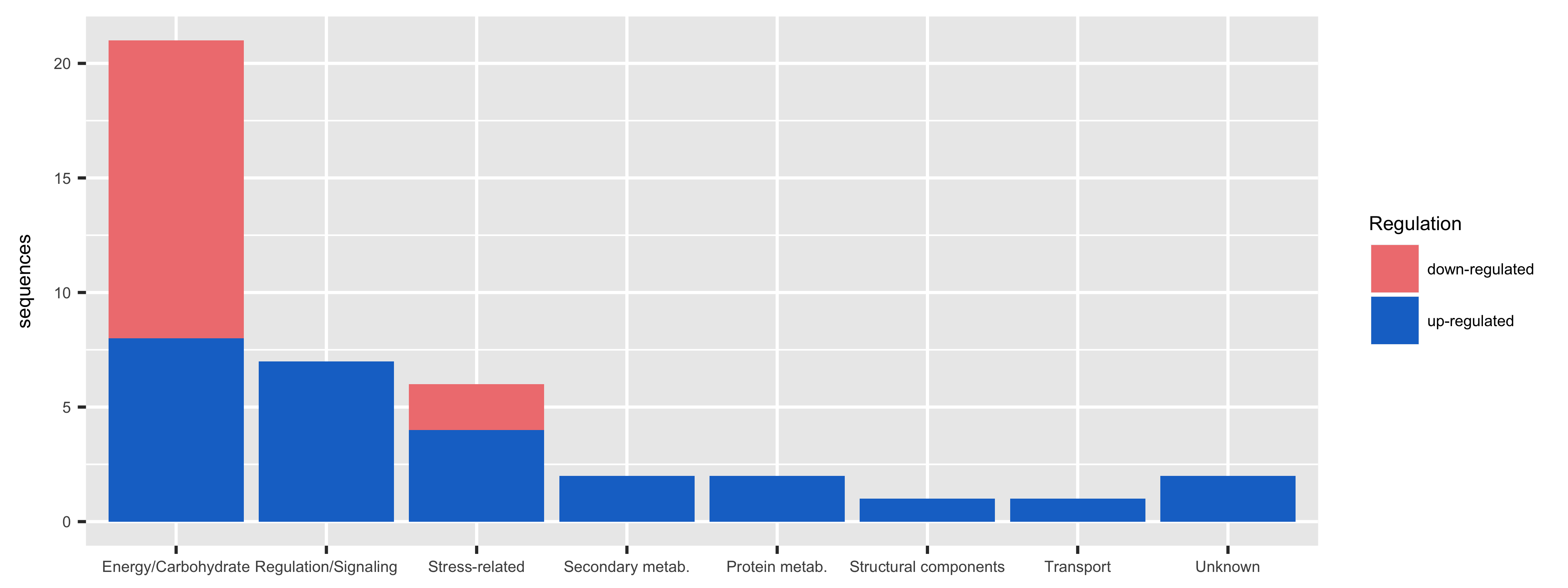

Supplement: S3 Fig — (TIFF) [file pone.0177389.s003.tiff]
