# Supplementary material for: Proteome dynamics of cold-acclimating Rhododendron species contrasting in their freezing tolerance and thermonasty behavior
Source: PLoS One. 2017 May 23;12(5):e0177389. doi: 10.1371/journal.pone.0177389 (PMC5441609; doi:10.1371/journal.pone.0177389)
Supplement: S2 Table — (DOCX) [file pone.0177389.s005.docx]

Table S2. Spots non-seasonally regulated in *R. catawbiense* during cold acclimation with significant higher (log_2_ expression ratio > 0) or lower levels (log_2_ expression ratio < 0) related to acclimated *R. ponticum* plants (PontCA).

| Spot Id. | Biological function/Pathway | Biological function/Pathway | CataCA/PontCA |
| --- | --- | --- | --- |
|  |  |  |  |
| Spot 67 | glutamine synthetase | General metabolism* | 4.74 |
| Spot 69 | plastid-lipid associated protein | Stress-related protein | 3.89 |
| Spot 49 | glutathione peroxidase | Oxidative stress protection | 1.85 |
|  |  |  |  |
| Spot 75 | glutamate-glyoxylate aminotransferase | General metabolism* | -2.28 |
| Spot 68 | ATP synthase gamma chain 2 | ATP synthesis, Energy metabolism | -2.70 |
| Spot 66 | glutamine synthetase | General metabolism* | -2.85 |
| Spot 64 | RuBisCO activase | Photosynthesis, Energy metabolism | -3.73 |
| Spot 70 | chlorophyll A/B binding protein | Photosynthesis, Photoinhibition | -4.17 |
| Spot 73 | ATP synthase CF1 alpha subunit | ATP synthesis, Energy metabolism | -4.71 |
| Spot 71 | oxygen-evolving enhancer protein | Photosynthesis, Photoinhibition | -4.88 |
| Spot 63 | UDP-galactose/ UDP-glucose transporter | Transport | -5.37 |

***** Biosynthesis of amino acids, Carbohydrate metabolism, Energy metabolism, Amino acid metabolism.
